# Evodiamine protects against airway remodelling and inflammation in asthmatic rats by modulating the HMGB1/NF-κB/TLR-4 signalling pathway

**DOI:** 10.1080/13880209.2020.1871374

**Published:** 2021-02-12

**Authors:** Qiong Wang, Yubao Cui, Xufeng Wu, Junfang Wang

**Affiliations:** a Department of Clinical Laboratory, Wuxi People’s Hospital Affiliated to Nanjing Medical University, Jiangsu, China; b Department of Chinese Traditional Medicine, Wuxi People’s Hospital Affiliated to Nanjing Medical University, Jiangsu, China; c Department of Orthopaedics, Wuxi People’s Hospital Affiliated to Nanjing Medical University, Jiangsu, China

**Keywords:** Asthma, bronchioles, cytokine, lung

## Abstract

**Context:**

Evodiamine, which is isolated from *Evodia rutaecarpa* (Rutaceae), possess strong anti-inflammatory, immunomodulatory, and antibacterial properties.

**Objective:**

The protective effects of evodiamine in asthma were evaluated.

**Materials and methods:**

Thirty-two Sprague-Dawley (SD) rats were used, asthma was induced by injecting intraperitoneally with a mixture of Al(OH)_3_ (100 mg) and ovalbumin (OA; 1 mg/kg), further exposing them to a 2% OA aerosol for 1 week. All animals were divided into four groups: control, asthma, and evodiamine 40 and 80 mg/kg p.o. treated group. Serum levels of inflammatory cytokines, interferon gamma (IFN-γ), and immunoglobulin E (IgE) and infiltrations of inflammatory cells in the bronchoalveolar lavage fluid (BALF) of the animals were determined. The thickness of the smooth muscle layer and airway wall in the intact small bronchioles of asthmatic rats was examined as well.

**Results:**

Cytokine levels in the serum and BALF were lower in the evodiamine-treated group than in the asthma group. Evodiamine treatment reduced IgE and IFN-γ levels as well as the inflammatory cell infiltrate in the lung tissue of asthmatic rats. The thickness of the smooth muscle layer and airway wall of intact small bronchioles was less in the evodiamine-treated group than in the asthma group. Lower levels of TLR-4, MyD88, NF-κB, and HMGB1 mRNA in lung tissue were measured in the evodiamine-treated group than in the asthma group.

**Discussion and conclusion:**

The effect of evodiamine treatment protects the asthma, as evodiamine reduces airway inflammation and remodelling in the lung tissue by downregulating the HMGB1/NF-κB/TLR-4 pathway in asthma.

## Introduction

Asthma is a respiratory disorder diagnosed by pathophysiological symptoms such as hyper-responsiveness, obstruction, remodelling, and inflammation of the airway. Some 339 million people suffer from asthma worldwide (Dharmage et al. 2019). Among the physiological pathways involved in the development of asthma is an imbalance in Th1/Th2 (Durrant and Metzger 2010). Th2 activation enhances the production of interleukins, leading to the development of several pathological conditions, including allergies and asthma. Allergic asthma occurs in response to enhanced production immunoglobulin E (IgE) by B cells following an increased release of cytokines (Galli and Tsai 2012). NF-κB, a potent inflammatory mediator, enhances the production of cytokines in several disorders, including asthma (Liu et al. 2017). Phosphorylation of IκB activates the NF-κB p65 subunit, which is responsible for increased production of cytokines and promotion of the inflammatory cascade (Christian et al. 2016). In addition, HMGB1, an immune system protein involved in the regulation of cell survival and death, has been implicated in sepsis, lung injury, and asthma. HMGB1 binds to toll-like receptors (TLRs), which stimulates the release of cytokines through inflammatory cells (Qu et al. 2019). MyD88- or non-MyD88-dependent signalling is used by HMGB1 to trigger downstream signals by activating TLR-4, which enhances the secretion of cytokines via the NF-κB pathway (Azam et al. 2019).

Cytokines and activation of the inflammatory cascade contribute to both the allergic reaction and the remodelling of lung tissue. Airway remodelling in asthmatic patients is induced by increased infiltration of T lymphocytes (LYM) and eosinophils (EOS; Fehrenbach et al. 2017). The persistent hyper-responsiveness of the airway together with remodelling cause irreversible obstruction, which in turn depresses respiratory function. The medication currently available to treat asthma offers only symptomatic relief and has several limitations. Thus, new approaches to the management of asthma are needed. Several reports have suggested that asthma can be managed by targeting the remodelling and inflammation of the airway (Bergeron et al. 2010).

There is increasing interest in molecules of natural origin as therapeutic agents. Evodiamine, which is isolated from *Evodia rutaecarpa* (Rutaceae), is a traditional medicine in China for treating cardiovascular disorders, infection, inflammation, and obesity (Liao et al. 2011). It also exhibits anticancer activity by regulating the TGF-β1 and NF-κB pathways and thereby controls the growth of several types of cancer cells (Jiang and Hu 2009). Anti-inflammatory activity, including innate immunity against bacterial infection, and antiulcer activity have also been demonstrated for evodiamine via its regulation of the inflammatory cascade and inflammasomes (Li et al. 2019; Yu et al. 2013). Thus, the present study evaluated the protective effects of evodiamine against asthma.

## Materials and methods

### Animals

Sprague-Dawley rats weighing 180–225 g and housed under controlled conditions (temperature of 24 ± 3 °C and 60 ± 5% humidity) with a 12 h light/dark cycle were used in this study. All animal experiments were approved by the Institutional Animal Ethical Committee of Wuxi People’s Hospital Affiliated with Nanjing Medical University, China (IAEC/WPH-NMU/2019/25).

## Chemicals

Aluminium hydroxide [Al(OH)_3_], ovalbumin (OA) and evodiamine were procured from Sigma Aldrich Ltd., USA. ELISA kits were purchased from ThermoFisher Scientific Ltd., China and anti-NF-κB antibodies were purchased from Abcam Ltd., USA. Total RNA Kit was purchased from Omega Bio-Tek Inc., USA.

### Experimental design and treatment protocol

**Figure 1.**
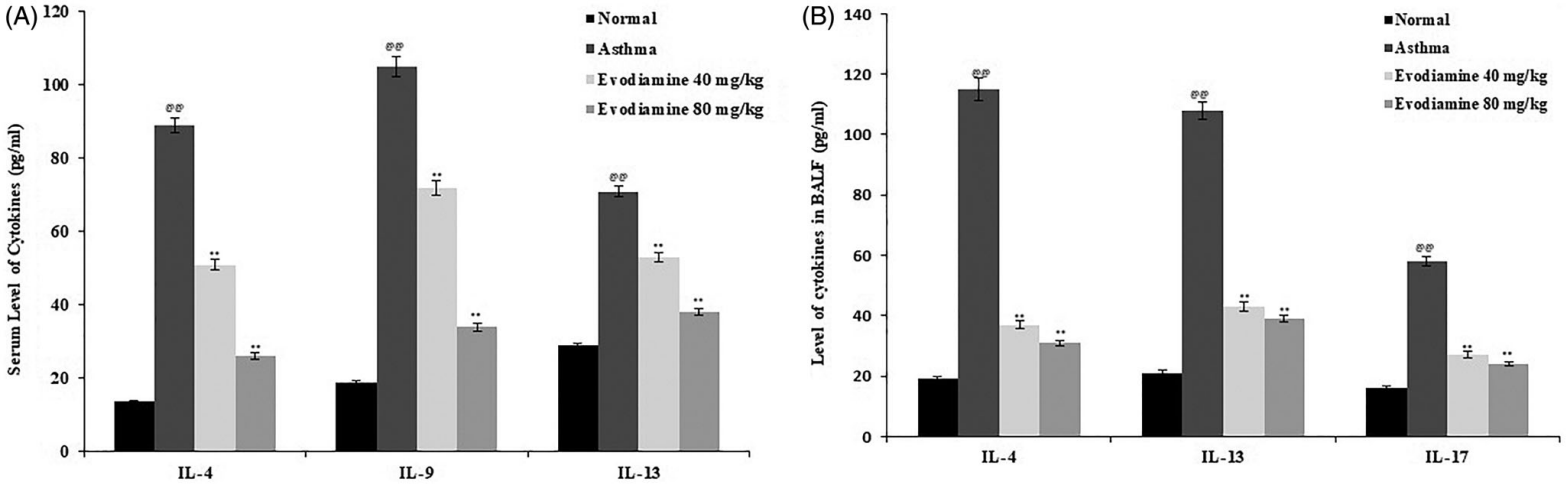
Influence of evodiamine on cytokine levels in the serum (A) and BALF (B) of asthmatic rats. Values are means ± SD (*n* = 8); ^@@^*p* < 0.01 compared to the normal group; **p < 0.01 compared to the asthma group.

We induced asthma by sensitising rats with an intraperitoneal injection of Al(OH)_3_ (100 mg) and ovalbumin (OA; 1 mg/kg). Fifteen days later, the rats were kept in an 0.8 m^3^ chamber, where they were exposed to 2% OA aerosol via an airflow of 8 L/min for 20 min/day for eight consecutive days. Rats were separated into four groups: a normal control group; an asthma group sensitised with OA but treated with normal saline solution; and two evodiamine groups, treated with 40 and 80 mg/kg group p.o (Shen et al. 2019). Evodiamine dose preparation was prepared by dissolving it in DMSO solution (Figure 1).

### Measurement of cytokines in the serum

Rats were anaesthetized at the end of the treatment protocol and blood was drawn. Then they were euthanized by intraperitoneal injection of sodium pentobarbital (100 mg/kg). Serum was separated, and serum levels of the cytokines interleukin (IL)-4, IL-9, and IL-13 were measured with ELISA kits per the manufacturer’s instructions.

### Preparation of BALF and determination of biochemical parameters

We ligated the right lung of each rat to determine histological changes and collected BALF from the left lung after cannulating the trachea. Bronchoalveolar lavage fluid (BALF) was centrifuged at 4 °C at 2500 rpm for 10 min. The pellet was resuspended in 100 µL PBS and used to determine relative and total leukocyte counts with a haemocytometer. Interferon gamma (IFN-γ), IgE, IL-4, IL-13, and IL-17 levels were measured in the supernatant with ELISA kits.

### Measurement of the thickness of smooth muscle and the airway wall

We examined isolated intact small bronchioles by light microscopy to measure the thickness of the airway wall and smooth muscle layer. Image-Pro Plus version 6.0 was used to determine the internal smooth muscle area (Wam1), external smooth muscle area (Wam2), bronchial luminal area (Wat1), total bronchial wall area (Wat2), and basement membrane perimeter (Pbm) in the bronchioles. The thicknesses of the smooth muscle layer (Wat) and airway wall (Wan) were calculated as follows:

$$\begin{array}{l} \text{Wan }=\;(\text{Wat1 }–\text{Wat2})/\text{Pbm}\\ \text{Wat }=\;(\text{Wam1 }–\text{Wam2})/\text{Pbm} \end{array}$$

### Histopathological analyses

Lung tissue was isolated from each animal, fixed in paraformaldehyde (4%), embedded in paraffin, and sectioned at a thickness of 5 µm. The sections were stained with H&E, and their histopathology was analysed with optical microscopy. The amount of inflammatory injury to the lung tissue was determined on a scale from 0 to 5: 0: normal, 1: few cells, 2: inflammatory cells that form a ring one layer deep, 3: a 2- to 4-cell layer of inflammatory cells, 4: a ring of inflammatory cells >4 cells deep.

We analysed the airway epithelium for goblet cells by staining it with periodic acid-Schiff (PAS) stain. Goblet cell hyperplasia was scored based on the percentage of PAS-positive cells: 0: no goblet cells, 1: <25% goblet cells, 2: 25–50%, 3: 51–75%, 4: >75%.

### Immunohistochemical analyses

Lung tissue was incubated overnight at 4 °C with anti-NF-κB antibodies and then with secondary antibody for 60 min at 37 °C. Image-Pro Plus version 6.0 was used to quantify the number of positive cells.

### qRT-PCR

The relative expression of TLR-4, MyD88, NF-κB, HMGB1, and GAPDH mRNA was estimated with SYBR green-based qRT-PCR. Total RNA was extracted with TRIzol reagent and then subjected to TaqMan MicroRNA assays. cDNA was synthesised from 2 µg total RNA (20 µL) with Moloney murine leukaemia virus reverse transcriptase. An ABI Prism 7500 system (Applied Biosystems, Foster City, CA, USA) was used with a SYBR green/fluorescein qPCR Master Mix kit (Thermo Fisher Scientific) with the following conditions: 50 °C for 2 min; 95 °C for 10 min; followed by 40 cycles at 95 °C for 30 s and 60 °C for 30 s, and a quantitative SYBR Green PCR assay was performed to estimate gene expression, calculated for each gene with the 2^-ΔΔCt^ method.

**Table:** 

Primer	Forward	Reverse
TLR-4	5′-CCCTGAGGCATTTAGGCAGCTA-3′	5′-AGGTAGAGAGGTGGCTTAGGCT-3′
MyD88	5′-GGCATTTCACTGCTTGATGT-3′	5′-TGACATTCCCATGAAACCTC-3′
NF-κB	5′-GCTACACAGAGGCCATTGAA-3′	5′-GTGGAGGAAGACGAGAGAGG-3′
HMGB1	5′-TTAGTCCCAGCGAAGGCTAT-3′	5′-CAAGTTTCCTGAGCAATCCA-3′
GAPDH	5′-GCAAGTTCAACGGCACAG-3′	5′-CGCCAGTAGACTCCACGAC-3′

### Homology model of TLR-4

We obtained the TLR-4 protein sequence from the NCBI database and used it to prepare a homology model using SWISS-Model server. UniProt was used to analysed the sequence of the TLR-4 protein. The target sequences were matched against the primary amino acid sequence in a BLAST search. The best quality templates were selected to build the homology model.

### Preparation of proteins and ligand and molecular docking

The 2 D structure of evodiamine was obtained from the PubChem database and converted into a .pdb file with Open Babel. AutoDock Vina MGL tools were used to prepare the ligand for the docking study by removing the water molecules, adding hydrogens, and modulating the charges according to the Kollman approach. A PDBQT file was thus obtained. We performed a molecular docking simulation of the ligand sophoridine using AutoDock Kollman and Gasteiger functions for both the ligand and the target protein. The grid map was created with AutoGrid 4. The grid box was prepared, and we defined the area of protein structure to be mapped by providing the coordinates. The grid box dimensions (x, y, and z coordinates) for TLR-4 were 111.168929, −8.862438, and −4.180014. The Lamarckian genetic algorithm was used for energy minimisation and optimisation in the docking simulation process.

### Statistical analyses

All data are expressed as means ± standard deviations (*n* = 8). The statistical analyses consisted of one-way analyses of variance (ANOVAs). *Post hoc* comparisons of the means were performed with Dunnett’s *post hoc* test in GraphPad Prism (ver. 6.1; San Diego, CA, USA). *p* < 0.05 was considered to indicate statistical significance.

## Results

### Evodiamine reduces the cytokine level in serum and BALF

Cytokine levels in the BALF and serum of evodiamine-treated asthmatic rats are shown in Figure 1(A,B). Serum IL-4, IL-9, and IL-13 levels were significantly (*p* < 0.01) enhanced in the asthma group compared with the control group. However, serum levels of the cytokines IL-4, IL-9, and IL-13 were reduced significantly (*p* < 0.01) in the evodiamine-treated group compared with the asthma group (Figure 1(A)). Moreover, levels of IL-4, IL-13, and IL-17 in BALF were increases in the asthma group relative to the normal group. Treatment of the asthmatic rats with evodiamine prevented this increase, with IL-4, IL-13, and IL-17 levels in BALF, relative to the asthma group (Figure 1(B)).

### Evodiamine reduces the thickness of smooth muscle and the airway wall

**Figure 2.**
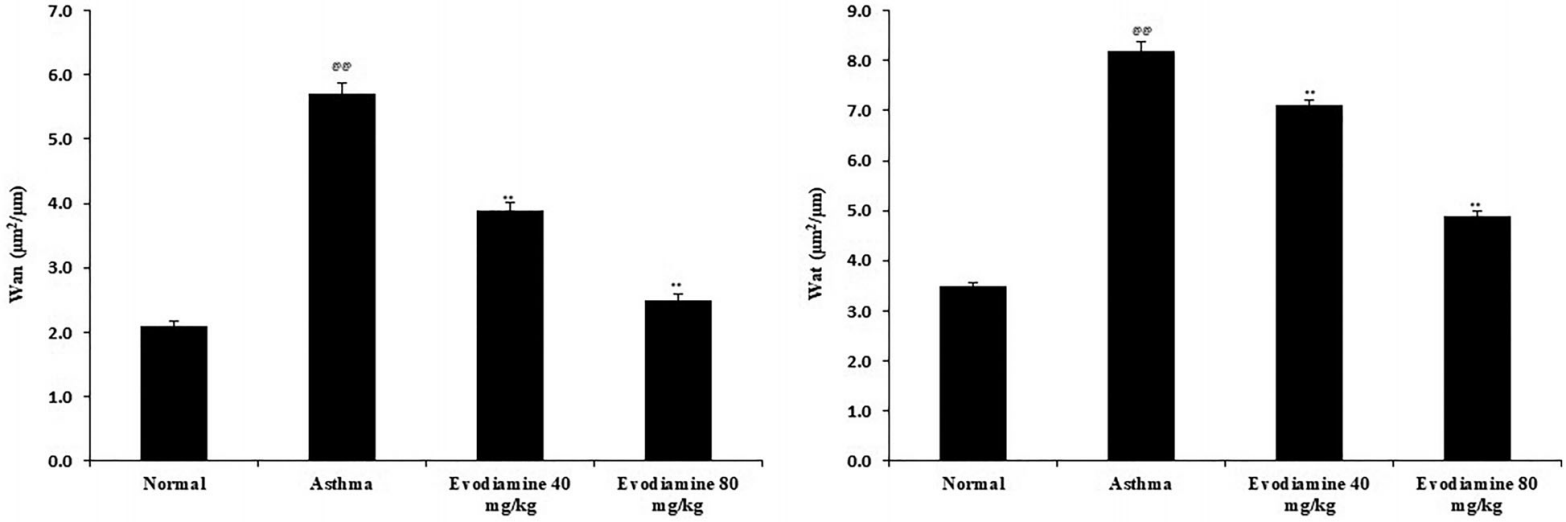
Evodiamine reduces the thickness of airway smooth muscle and the airway wall in intact small bronchioles of asthmatic rats. Values are means ± SD (*n* = 8); ^@@^ *p* < 0.01 compared to the normal group; ** *p* < 0.01 compared to the asthma group.

Isolated intact small bronchioles of evodiamine-treated and untreated asthmatic rats are shown in Figure 2. Thickness of the airway wall and smooth muscle layer was enhanced in the lung tissue of asthma group than control group of rats. However, treatment with evodiamine significantly (*p* < 0.01) reduces the thickness of both (airway wall and smooth muscle) in the lung tissue of asthmatic rats.

### Evodiamine attenuates biochemical parameters

**Figure 3.**
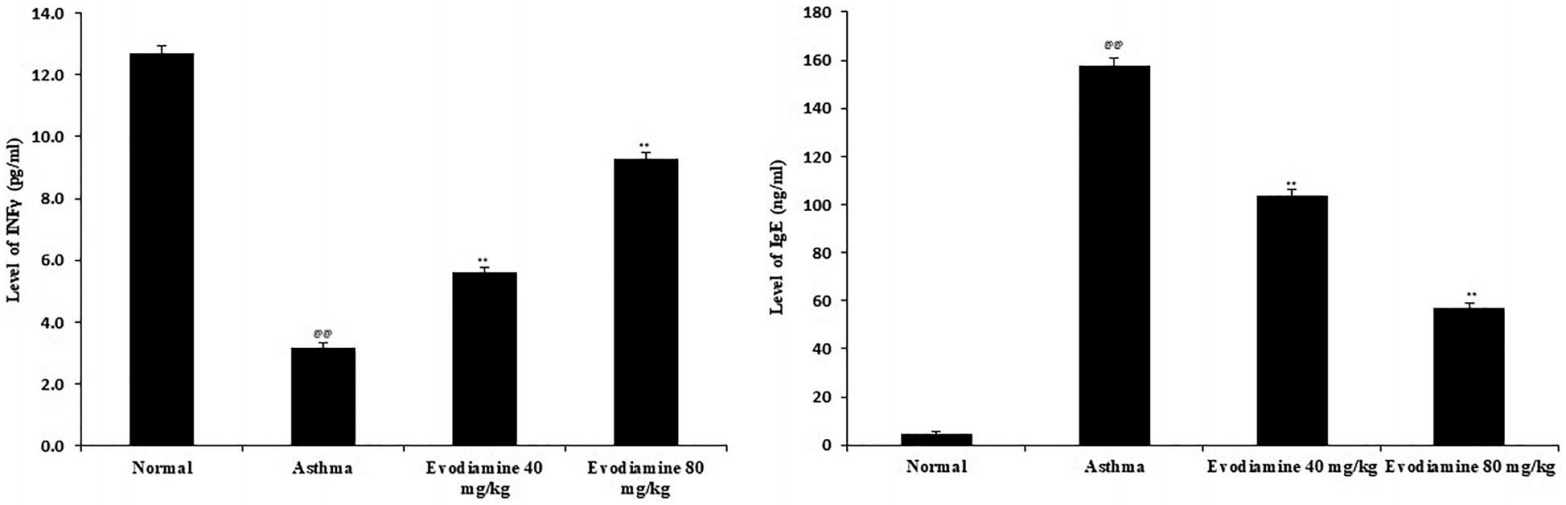
Effects of evodiamine on IFN-γ and IgE levels in lung tissue homogenates of asthmatic rats. Values are means ± SD (*n* = 8); ^@@^*p* < 0.01 compared to the normal group; **p < 0.01 compared to the asthma group.

Levels of mediators that modulate the inflammatory reaction, including IFN-γ and IgE, were measured in lung tissue homogenates. In the lung tissue of asthmatic rats, IFN-γ decreased significantly (*p* < 0.01) and increase in IgE level than normal control group. Evodiamine treatment increased the level of IFN-γ and significantly reduces the level of IgE in the lung tissue compared to asthma group of rats (Figure 3).

### Evodiamine attenuates the infiltration of inflammatory cells

**Figure 4.**
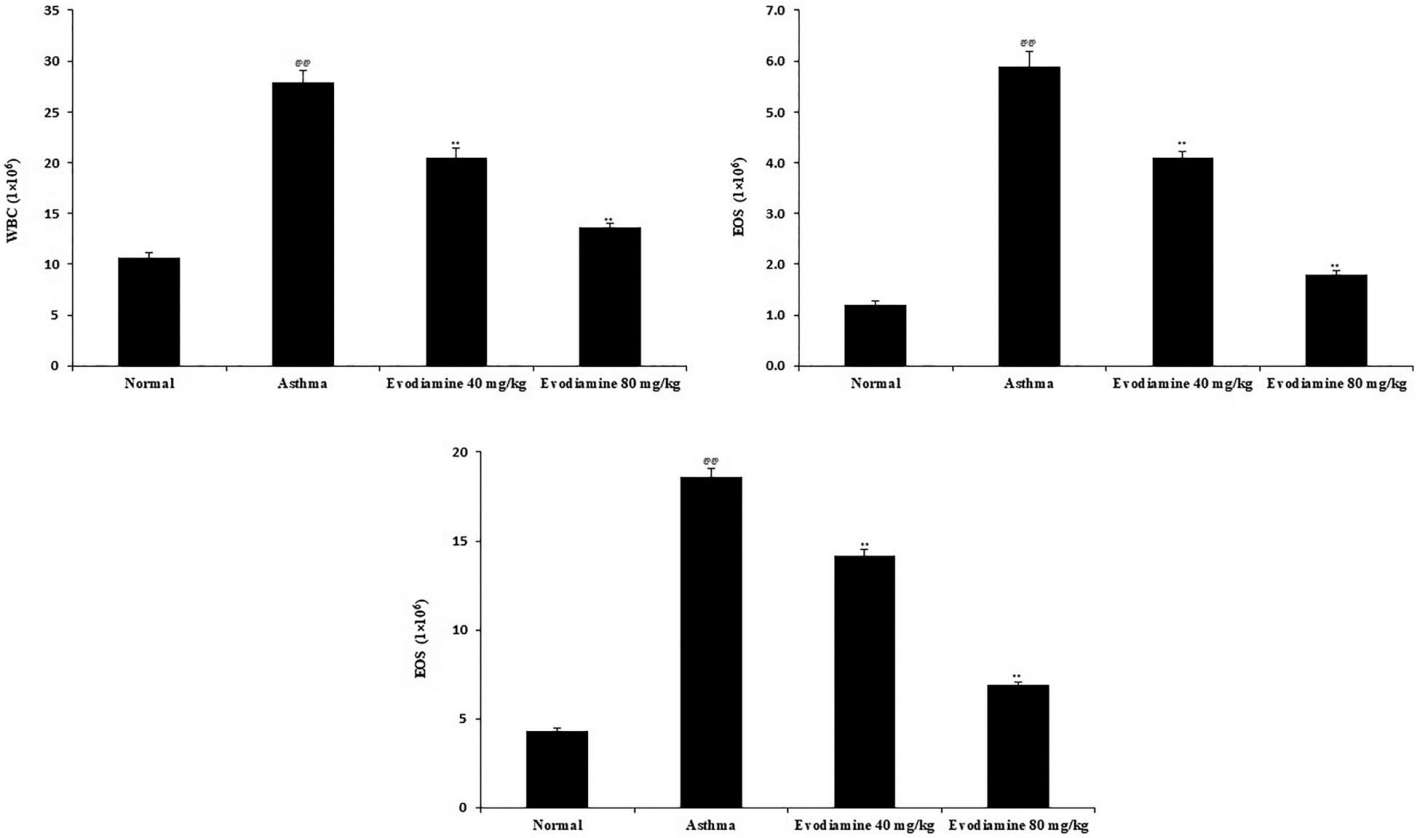
Effects of evodiamine on the infiltration of inflammatory cells in the BALF of asthmatic rats. Values are means ± SD (*n* = 8); ^@@^*p* < 0.01 compared to the normal group; **p < 0.01 compared to the asthma group.

We evaluated the infiltration of inflammatory cells by determining the level of white blood cells (WBC), EOS, and LYM in the BALF of the four groups of rats (Figure 4). WBC, EOS, and LYM counts were higher in the BALF of the asthma group than the normal group of rats, whereas they decreased significantly (*p* < 0.01) in the BALF of evodiamine-treated asthmatic rats.

### Evodiamine attenuates expression of NF-κB and HMGB1 protein

**Figure 5.**
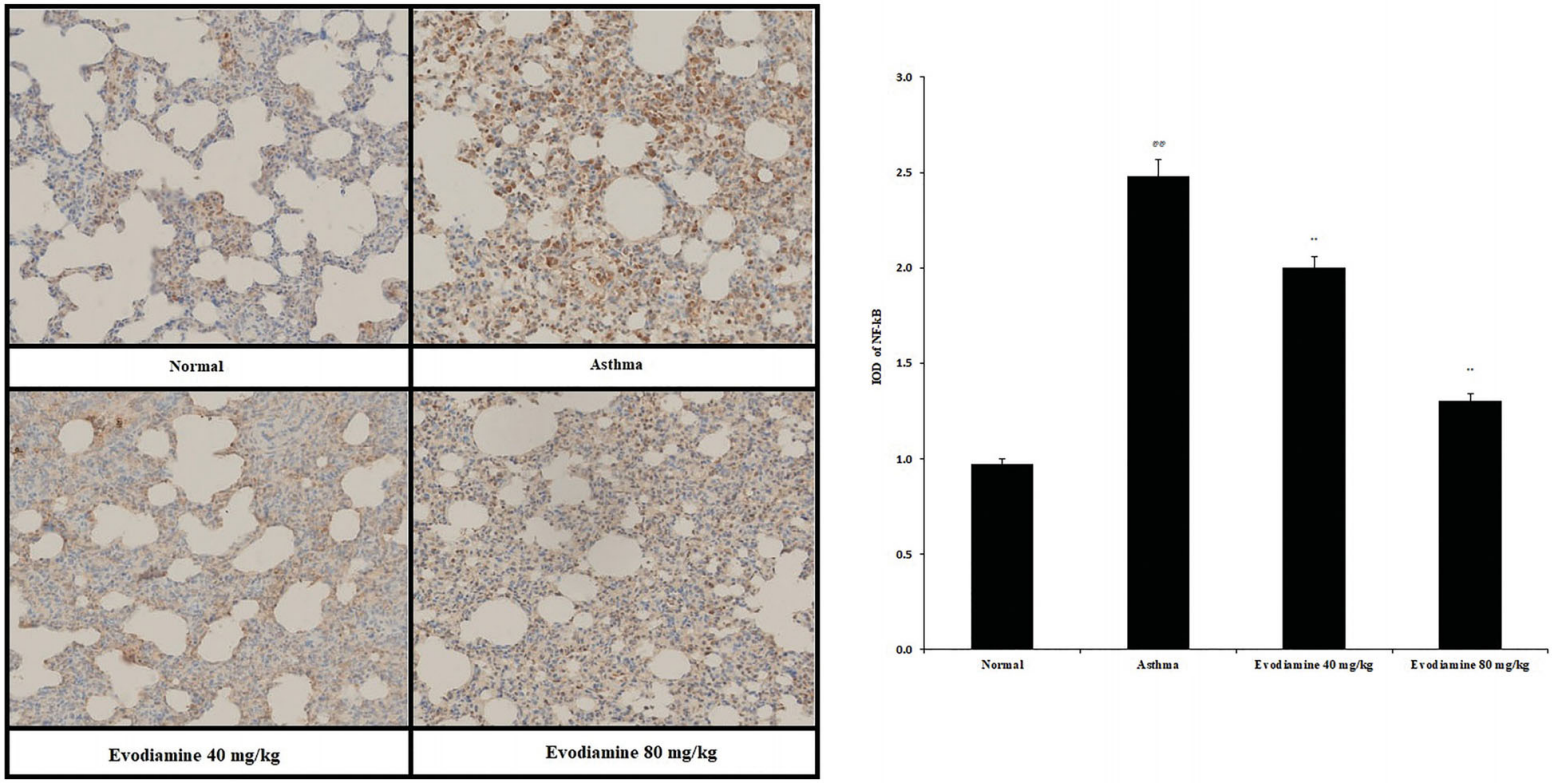
Immunohistochemical analyses of the expression of NF-κB protein in the lung tissue of asthmatic rats. Values are means ± SD (*n* = 8); ^@@^*p* < 0.01 compared to the normal group; **p < 0.01 compared to the asthma group.

Expression of NF-κB protein in the lung tissue of the rats is shown in Figure 5(A,B). Expression was higher in the asthma group than in the normal group, but it was reduced following treatment with evodiamine.

### Evodiamine attenuates mRNA expression of TLR-4, MyD88, NF-κB, and HMGB1

**Figure 6.**
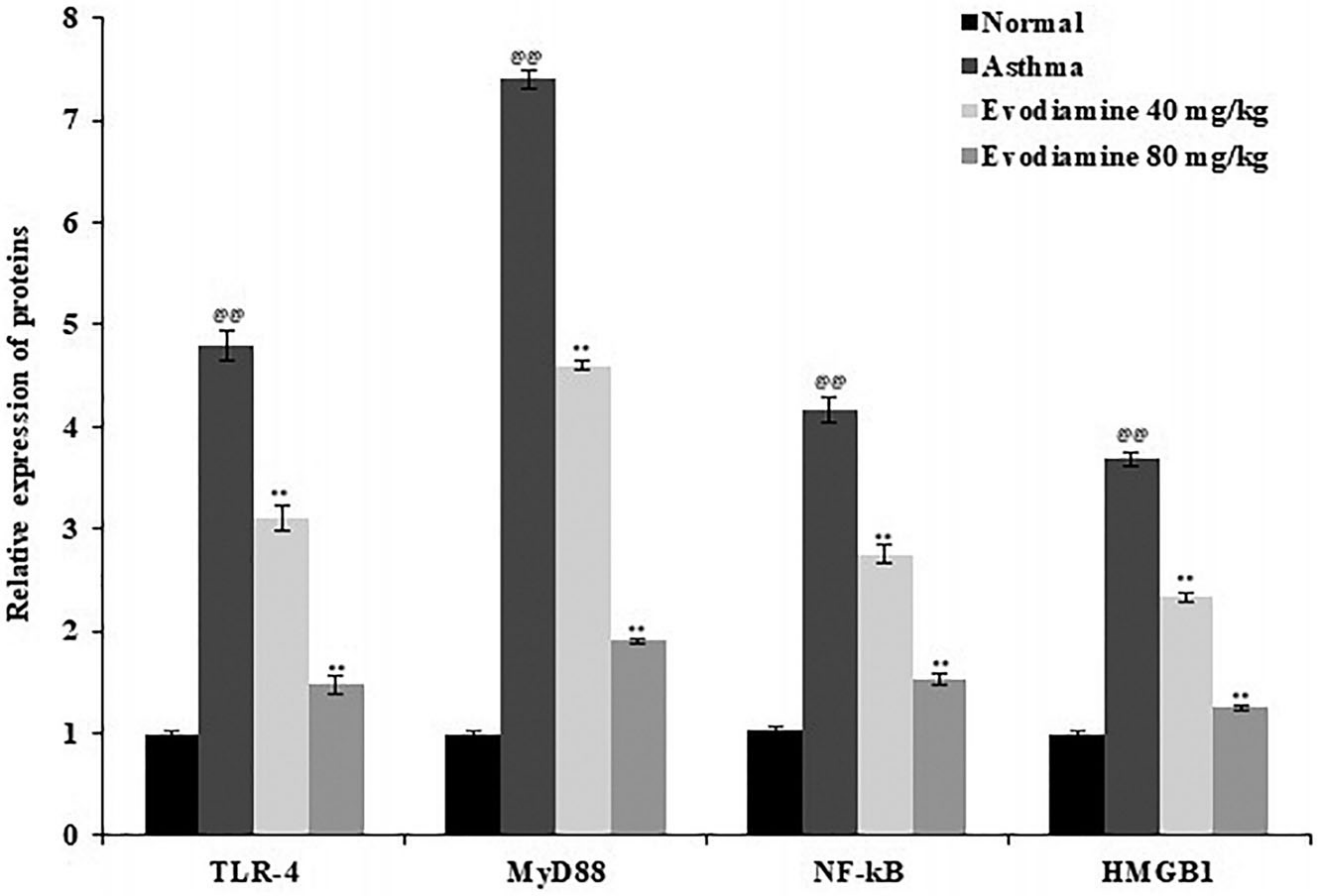
Expression of TLR-4, MyD88, NF-κB, and HMGB1 mRNA in the lung tissue of evodiamine-treated asthmatic rats. Values are means ± SD (*n* = 8); ^@@^*p* < 0.01 compared to the normal group; **p < 0.01 compared to the asthma group.

TLR-4, MyD88, NF-κB, and HMGB1 mRNA expression was measured in rat lung tissue (Figure 6). Levels of all four mRNAs were significantly enhanced in the lung tissue of the asthma group compared to the normal group but were reduced in the lung tissue of the evodiamine-treated group compared to the untreated asthmatic group.

### Evodiamine attenuates histopathological changes in lung tissue

**Figure 7.**
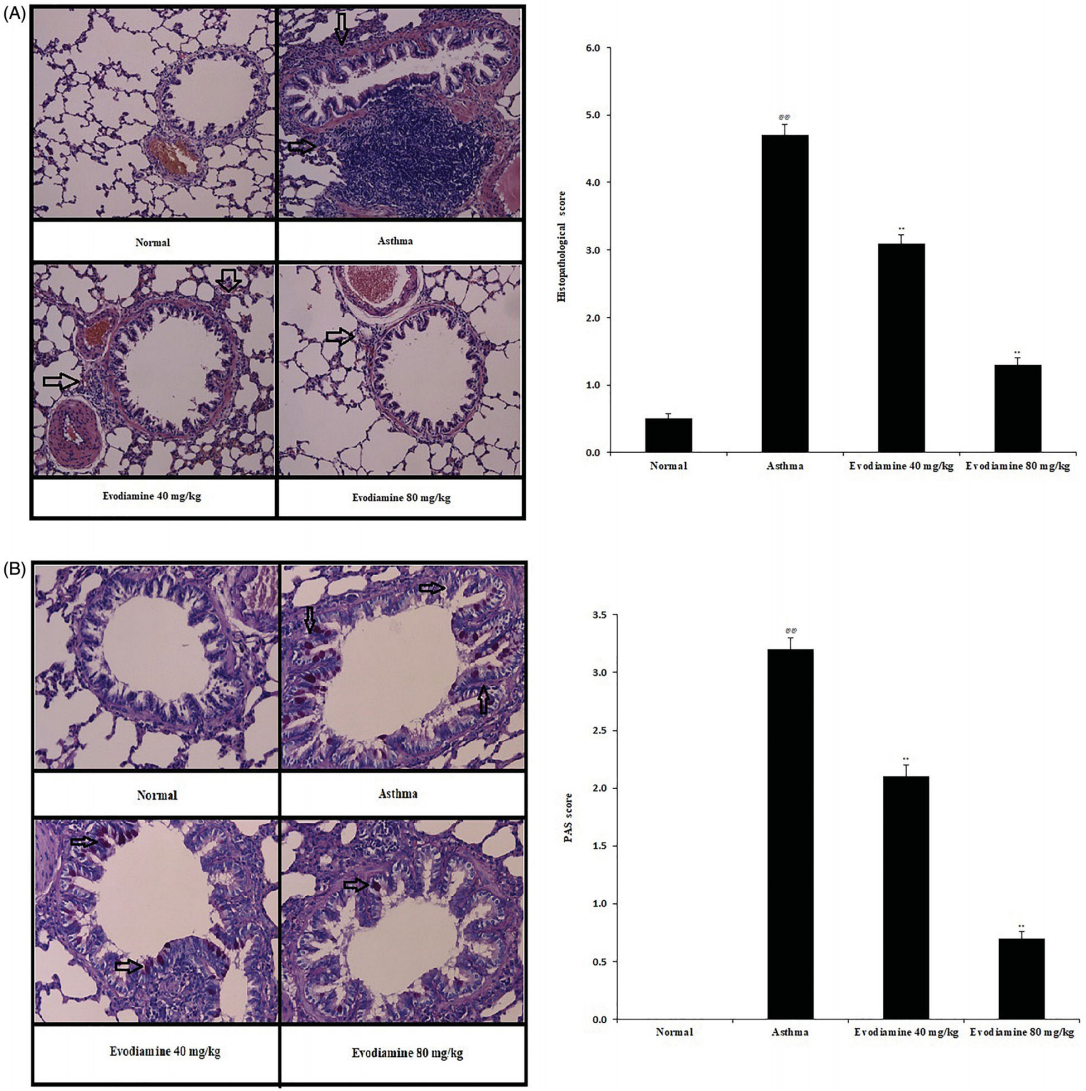
Histopathological changes in the lung tissue of evodiamine-treated asthmatic rats. (A) H&E staining of lung tissue and histopathological score. (B) PAS staining of lung tissue and PAS score. Values are means ± SD (*n* = 8); ^@@^*p* < 0.01 compared to the normal group; **p < 0.01 compared to the asthma group.

Histopathological changes in the lung tissue of rats treated with evodiamine or not treated are shown in Figure 7(A,B). Inflammation scores were estimated based on histopathological scores determined from H&E-stained sections (Figure 7(A)). The histopathological score was higher in the asthma group than in the normal group, but the increase was reversed by treatment with evodiamine.

The number of goblet cells in lung tissue was estimated by PAS staining (Figure 7(B)). We found a higher PAS score in the asthma group than in the normal group but a dose-dependent reduction in PAS score in the evodiamine-treated group versus the asthma group.

### Effects of evodiamine on the TLR-4 protein

**Figure 8.**
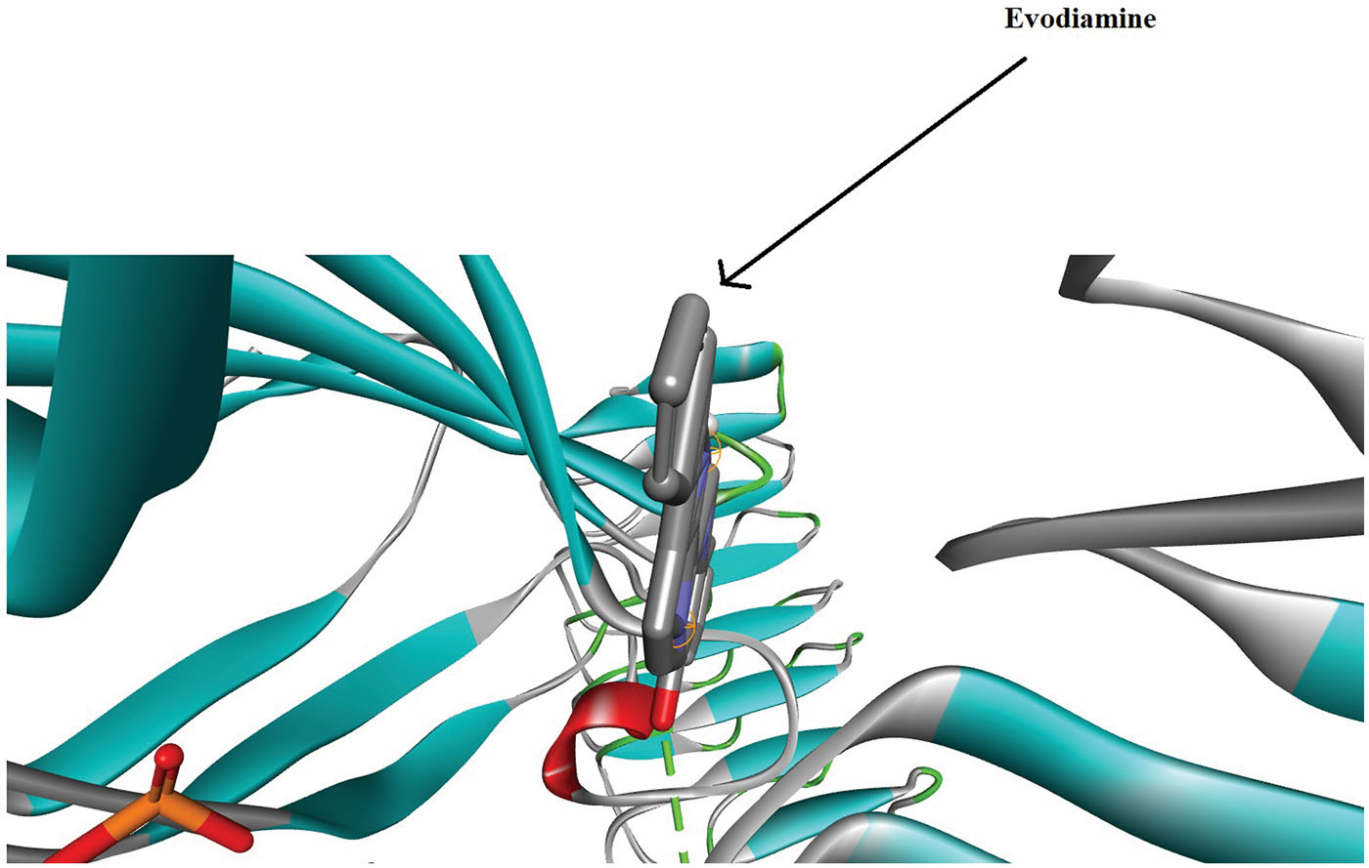
*In silico* molecular docking shows the interaction of the TLR-4 protein and evodiamine. The solid area in the protein structures represents the area of interaction.

Given the *in vivo* and *in vitro* findings, we performed a molecular docking study using BLAST and homology modelling followed by ligand and protein preparation. Molecular docking simulation of the ligand evodiamine was done with the AutoDock Kollman and Gasteiger functions for both the ligand and the binding protein. The docking results showed the high binding affinity of evodiamine with the *in vivo* confirmed TLR-4 protein, a result supported by the high binding energies (Table 1). The 3 D structure of the protein revealed the area of ligand binding in the TLR-4 protein (Figure 8).

**Table 1.** Docking scores for TLR-4 protein with ligand molecule Evodiamine.

Docking Score for TLR-4 with ligand Evodiamine			
Mode of ligand	Affinity (kcal/mol)	Distance from rmsd l.b	Best mode rmsd u.b
1	−8.9	0.000	0.000
2	−8.8	13.120	16.496
3	−8.8	2.930	3.627
4	−8.7	1.049	2.163
5	−8.7	12.131	13.776
6	−8.6	11.944	14.646
7	−8.6	12.283	15.506
8	−8.6	11.870	14.942
9	−8.5	8.502	9.972

## Discussion

Asthma is a chronic disorder characterised by hyper-responsiveness of the bronchial tree due to inflammatory responses in the lung. Several pathophysiological pathways are associated with the development of asthma, including those that mediate the release of cytokines involved in the inflammatory process, in particular IL-4, IL-9, IL-13, and IL-17. Although leukotriene inhibitors have shown potential in the management of asthma, our study demonstrates that treatment with evodiamine ameliorates enhanced levels of cytokines in the serum and BALF of asthmatic rats.

The infiltration of inflammatory cells in the lung is another feature of asthma (National Asthma Education and Prevention Program, Third Expert Panel on the Diagnosis and Management of Asthma 2007). These inflammatory cells are responsible for the stimulated release of inflammatory cytokines and their subsequent effects on the smooth muscle of the respiratory tract (Moldoveanu et al. 2009). IgE levels are also enhanced in asthmatic patients and are responsible for inducing allergic reaction by releasing histamine (Yamauchi and Ogasawara 2019), a tissue amine responsible for the increased sensitivity of the bronchial tree and the contraction of its smooth muscle layer (Bonaldi et al. 2003). In our rat model of asthma, evodiamine ameliorated the altered level of IgE and reduced infiltration of inflammatory cells in the lung tissue of asthmatic rats. In addition, evodiamine reduced the thickness of both the airway wall and the smooth muscle layer in treated rats compared to untreated asthmatic rats.

Reports suggest that inflammation enhances the permeability of the nuclear membrane, allowing the nuclear HMGB1 protein to reach the cytoplasm (Sprague and Khalil 2009). Leukotrienes are secreted by macrophages and mononuclear cells, and their release is stimulated by HMGB1 (Baek et al. 2018). The knockdown of HMGB1 reduces the severity of asthma by reducing airway smooth muscle thickness, collagen deposition, mucus secretion, and inflammation in the airway (Hou et al. 2015). The TLR-4 protein, the receptor for HMGB1, is involved in the immune cell response and in inflammation, and its overexpression in the lung enhances the thickness of the airway wall by activating elastase and NF-κB (Wang et al. 2020). The MyD88-dependent pathway is used by TLR-4 to activate NF-κB and further contributes to the synthesis of inflammatory cytokines (Liu et al. 2017). Our study suggests a mechanism by which evodiamine hinders the development of asthma in rats by altering expression of TLR-4, NF-κB, MyD88, and HMGB1, thus hindering airway remodelling in the rat lung.

## Conclusion

This reports suggests that treatment with evodiamine reduces inflammation and airway remodelling in the lung tissue of asthmatic rats by downregulating the HMGB1/NF-κB/TLR-4 pathway. Data of the study reveal that evodiamine could be explored clinically for the management of asthma.
